# A proliferation saturation index to predict radiation response and personalize radiotherapy fractionation

**DOI:** 10.1186/s13014-015-0465-x

**Published:** 2015-07-31

**Authors:** Sotiris Prokopiou, Eduardo G. Moros, Jan Poleszczuk, Jimmy Caudell, Javier F. Torres-Roca, Kujtim Latifi, Jae K. Lee, Robert Myerson, Louis B. Harrison, Heiko Enderling

**Affiliations:** Departments of Integrated Mathematical Oncology, H. Lee Moffitt Cancer Center & Research Institute, 12902 Magnolia Drive, Tampa, FL 33612 USA; Department of Radiation Oncology, H. Lee Moffitt Cancer Center & Research Institute, 12902 Magnolia Drive, Tampa, FL 33612 USA; Department of Cancer Imaging and Metabolism, H. Lee Moffitt Cancer Center & Research Institute, 12902 Magnolia Drive, Tampa, FL 33612 USA; Department of Biostatistics and Bioinformatics, H. Lee Moffitt Cancer Center & Research Institute, 12902 Magnolia Drive, Tampa, FL USA; Department of Radiation Oncology, Washington University School of Medicine, St. Louis, MO USA

**Keywords:** Proliferation saturation index, Personalized radiotherapy, Logistic tumor growth, Mathematical model, Hyperfractionation

## Abstract

**Background:**

Although altered protocols that challenge conventional radiation fractionation have been tested in prospective clinical trials, we still have limited understanding of how to select the most appropriate fractionation schedule for individual patients. Currently, the prescription of definitive radiotherapy is based on the primary site and stage, without regard to patient-specific tumor or host factors that may influence outcome. We hypothesize that the proportion of radiosensitive proliferating cells is dependent on the saturation of the tumor carrying capacity. This may serve as a prognostic factor for personalized radiotherapy (RT) fractionation.

**Methods:**

We introduce a proliferation saturation index (PSI), which is defined as the ratio of tumor volume to the host-influenced tumor carrying capacity. Carrying capacity is as a conceptual measure of the maximum volume that can be supported by the current tumor environment including oxygen and nutrient availability, immune surveillance and acidity. PSI is estimated from two temporally separated routine pre-radiotherapy computed tomography scans and a deterministic logistic tumor growth model. We introduce the patient-specific pre-treatment PSI into a model of tumor growth and radiotherapy response, and fit the model to retrospective data of four non-small cell lung cancer patients treated exclusively with standard fractionation. We then simulate both a clinical trial hyperfractionation protocol and daily fractionations, with equal biologically effective dose, to compare tumor volume reduction as a function of pretreatment PSI.

**Results:**

With tumor doubling time and radiosensitivity assumed constant across patients, a patient-specific pretreatment PSI is sufficient to fit individual patient response data (R^2^ = 0.98). PSI varies greatly between patients (coefficient of variation >128 %) and correlates inversely with radiotherapy response. For this study, our simulations suggest that only patients with intermediate PSI (0.45–0.9) are likely to truly benefit from hyperfractionation. For up to 20 % uncertainties in tumor growth rate, radiosensitivity, and noise in radiological data, the absolute estimation error of pretreatment PSI is <10 % for more than 75 % of patients.

**Conclusions:**

Routine radiological images can be used to calculate individual PSI, which may serve as a prognostic factor for radiation response. This provides a new paradigm and rationale to select personalized RT dose-fractionation.

## Background

Recent advances in radiation oncology have largely focused on the physical characteristics of radiation including beam quality and delivery. Typically, tumor and normal tissue anatomy/geometry are only used as the main parameters with which to enhance therapeutic ratios [1–5]. Adaptive radiotherapy and image-guided radiotherapy have been suggested to primarily re-shape the target volume based on changes in tumor volume or position [1–4], rather than as a methodology to adapt to changes in the intrinsic tumor-host biology. Inroads have been made in combining intensity-modulated radiation therapy with functional imaging, such as by [^18^F]-fluoromisonidazole positron emission tomography (FMISO-PET) or dynamic contrast-enhanced magnetic resonance imaging, for radiation planning and response assessment to direct higher doses of radiation to areas with increased radioresistance [5–8]. In conventional clinical practice, however, most patients treated with definitive radiotherapy receive a similar dose and fractionation scheme based upon primary site and American Joint Committee on Cancer (AJCC) TNM stage (Tumor size, lymph Node involvement, Metastasis presence).

Different fractionation protocols have been tested in prospective clinical trials [9, 10]. Alternative radiation fractionation protocols may improve outcome for some patients but worsen outcome for others. It is important that we begin to understand which tumors respond better to altered fractionation, and how to select the most appropriate fractionation schedule for an individual patient. Innovative models that are based upon cell biology and interactions of the tumor with its unique environment could forecast individual radiation response and justify recommendation of either standard of care or alternative radiation fractionation on a per patient basis.

Tumors grow within a host tissue that both facilitates progression by supplying nutrients and growth factors [11, 12], and inhibits it through physical constraints [13] and immune surveillance [14, 15]. Since many of these factors vary widely across patients, we introduce the concept of tumor carrying capacity as the maximum tumor volume that is achievable in the patient-specific tumor environment, and saturation of tumor proliferation as the tumor approaches its carrying capacity. We propose a non-invasive radiomics measurement of patient-specific carrying capacity and proliferation saturation, which may ultimately help designing more personalized approaches to radiotherapy.

Tumors are composites of proliferating and growth arrested cells. Their respective proportions at individual times during radiation contribute to the population-level response, in addition to radiation beam and protocol parameters and host tissue properties. In multi-compartment mathematical models that distinguish between cycling and growth-arrested cells, proliferation and oxygenation status-dependent radiation response could be simulated on the cellular level [16, 17]. Tumor growth *in vivo* can be approximated by logistic dynamics [18]. Initial exponential growth at low cell densities when most cells have access to ample resources decelerates when cells at the core of the tumor become growth-arrested, mainly due to limited space and exhausted intratumoral nutrient supply as resources are consumed by cells closer to the tumor surface [19, 20]. This established the notion of a tumor carrying capacity (K) as the maximum tumor volume (V) that can be supported by a given environment. A tumor carrying capacity may change depending on the oxygen and nutrient supply through tissue vascularization [21], removal of metabolic waste products [22], and evasion of immune surveillance [14]. Greater oxygen supply and removal of metabolic waste increases tumor carrying capacity; in contrast, infiltration of tumor specific cytotoxic T lymphocytes exemplifies a reduction of carrying capacity. Hence, the tumor volume-to-carrying capacity ratio (V/K) describes the saturation of tumor cell proliferation at the population level as the tumor approaches its carrying capacity, and as such is deemed the Proliferation Saturation Index (PSI). The PSI at any time reflects the history of the reciprocal changes of a tumor and its environment – and thus can be expected to be patient-specific. Tumor volumes close to their carrying capacity, i.e., with a high PSI, are here assumed to have only a small proportion of proliferating cells that are most sensitive to radiation-induced damage. We therefore hypothesize that a patient-specific PSI may serve as a novel prognostic factor for radiotherapy response.

## Methods

### Tumor radiation response model and Proliferation Saturation Index (PSI)

**Fig. 1:**
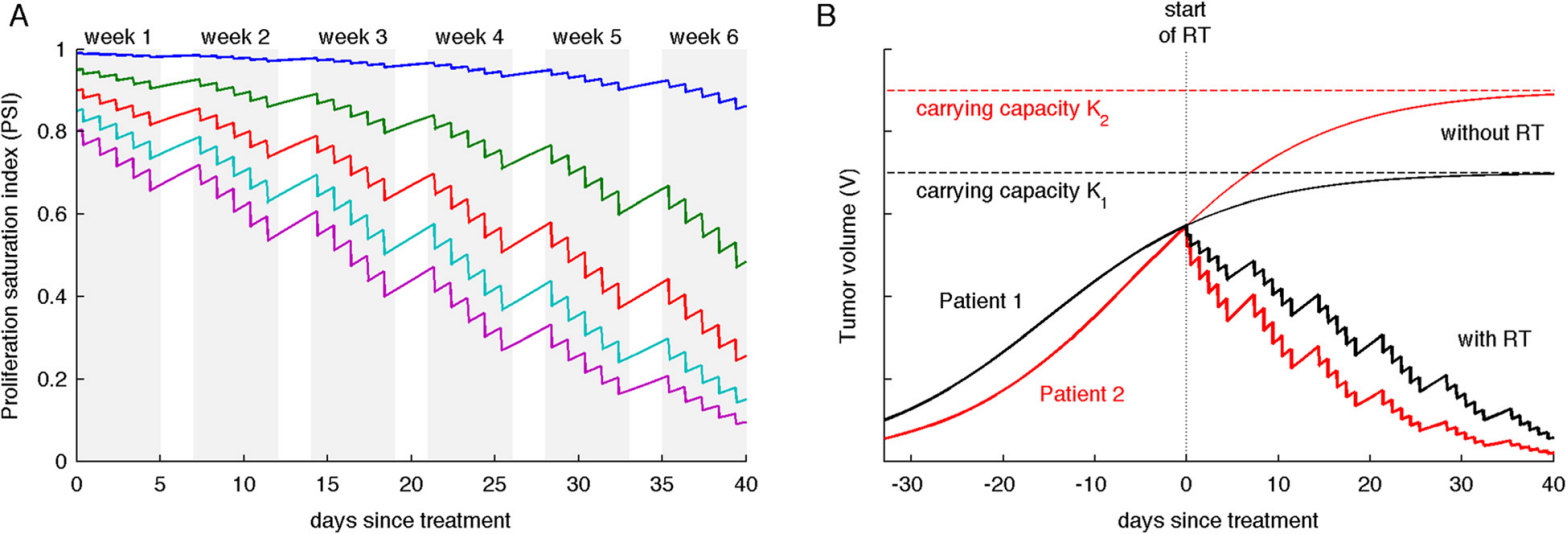
Pretreatment proliferation saturation index (PSI) determines RT response. **a** Response for tumors with different initial PSIs to standard of care RT (2Gy x 30; q.d. 9 am, no weekend) calculated with Eqn. 1; λ=0.1, γ_2Gy_=0.25. Each curve shows the dynamics response during six weeks of therapy with different initial PSI. **b** Two patients with identical tumor volume but different PSI at treatment beginning (day 0) exhibit different reduction in tumor volume after standard of care RT (2Gy x 30; q.d. 9 am, no weekend). Calculated with Eqn. 1; λ=0.1, γ_2Gy_=0.25

Logistic tumor growth is modeled as a deterministic ordinary differential equation between radiation doses

1 $$ \frac{\mathrm{dV}}{\mathrm{dt}}=\uplambda \mathrm{V}\left(1{}^{\circ}-{}^{\circ}\mathrm{P}\mathrm{S}\mathrm{I}\right) $$

where PSI is the tumor volume-to-carrying capacity ratio (V/K), and $\uplambda =\frac{ \ln 2}{{\mathrm{T}}_{\mathrm{eff}}}$ is the intrinsic tumor growth rate, with the effective tumor doubling time T_eff_ being a composite of the potential doubling time, T_POT_, reduced by the cell loss fraction, φ [23]. T_eff_ is assumed to be intrinsic and time independent. Radiation response after each application of a single dose *d* is modeled as an instantaneous volume change ${\mathrm{V}}_{\mathrm{postIR}}=\mathrm{V}-{\upgamma}_d\;\mathrm{V}\left(1-\frac{\mathrm{V}}{\mathrm{K}}\right)$ at discrete times during irradiation in the considered treatment protocols (standard of care: once a day [q.d.] at 9 am, no treatment on the weekend; hyperfractionation twice a day [b.i.d] at 9 am and 3 pm; no weekend), where ${\upgamma}_d=1-{e}^{-\left(\upalpha {\mathrm{d}}^{\circ }{+}^{\circ}\upbeta {\mathrm{d}}^2\right)}$ represents radiation-induced death following the linear-quadratic model [24, 25]. In this form, radiation-induced cell death is only considered for proliferating cells, while quiescent and hypoxic cells are assumed radioresistant. Tumor growth is modeled following (Eqn. 1) between radiation fractions. While other biological effects are without doubt at play before, during and after RT, longitudinal measurements of these effects are currently impossible and thus intentionally not considered explicitly. However, changes in tumor growth rate such as accelerated repopulation as well as other proliferation stimulating radiation effects (re-oxygenation, re-distribution in the cell cycle) are inherent to the logistic growth and RT model: as the tumor volume shrinks, V/K and thus PSI decreases, which in turn reduces the extrinsically enforced pre-treatment reduction in proliferation. It follows from (Eqn. 1) that larger PSI implies a low proliferating cell fraction and thus treatment refractory tumors, whereas tumors with lower PSI are more proliferative and thus more radiosensitive (Fig. 1a), which is in line with the established positive correlation between proliferation rate and radiosensitivity [18]. Therefore, two patients that present with similar tumor volume could have a different tumor environmental conditions and thus different PSI, which results in different responses to the same RT protocol (Fig. 1b).

Here we assume that the tumor carrying capacity is constant during the 5–7 week course of treatment. This is, of course, a gross oversimplification of the underlying biology, but without longitudinal measurements of carrying capacity biomarkers, calibration of dynamic carrying capacity changes during RT is impossible and would introduce additional uncertainty. The model was simulated in MATLAB using the analytical expression for the solution of (Eqn. 1) (MATLAB R2013b, The MathWorks Inc., Natick, MA).

### Prospective estimation of patient-specific pretreatment PSI

The analytical solution of pretreatment tumor growth (Eqn. 1) is given by $\mathrm{V}=\frac{\mathrm{K}\times \mathrm{V}(0)\times {\mathrm{e}}^{\lambda \mathrm{t}}}{\mathrm{K}+\mathrm{V}(0)\times \left({\mathrm{e}}^{\lambda \mathrm{t}}-1\right)}$, with V(0) being the initial tumor volume at time t=0. From two different radiological scans, routinely taken at diagnosis and at radiotherapy treatment planning simulation (Δt=45 days in private hospitals [26]), we obtain two distinct tumor volumes (V(0) =V_diagnosis_ and V=V_simulation_) along the logistic growth trajectory. Then, the analytical solution of the logistic model can be solved explicitly for K, giving the following analytic expression for pretreatment PSI value.

2 $$ \mathrm{P}\mathrm{S}\mathrm{I}=\frac{{\mathrm{V}}_{\mathrm{diagnosis}}\times {\mathrm{e}}^{\uplambda \varDelta \mathrm{t}}-\mathrm{Vsimulation}}{{\mathrm{V}}_{\mathrm{diagnosis}}\times \left({\mathrm{e}}^{\uplambda \varDelta \mathrm{t}}-1\right)} $$

### Data fitting

Thirteen longitudinal tumor volume measurements across four NSCLC patients treated with fractionated RT (2 Gy x 30 fractions) were taken from the literature [27]. Tumor volumes are available at the beginning of treatment (V_simulation_={7.6, 27.4, 97.7, 189.3} cm^3^) and at least two subsequent RT fractionations for each patient. We assume that the variations in intrinsic effective tumor growth rate $\lambda$ and radiosensitivity γ_2Gy_ are negligible between patients compared to the variation in individual carrying capacity K, given the spread of tumor volumes across two or three orders of magnitude. We therefore estimate constant λ and γ_2Gy_ values for all patients, and individual pretreatment proliferation saturation indices (PSI_s_).

We utilize a genetic algorithm that mimics the processes of evolution and natural selection [28] to derive a combination of parameters that best fits patient data. We generate an initial ‘population’ (*N*=500 ‘individuals’) of parameter sets {$\lambda$, γ_2Gy_, PSI_1_, PSI_2_*,* PSI_3_*,* PSI_4_}, with each element of each individual parameter set drawn at random from a uniform distribution [0,1]. In each algorithm iteration (‘generation’) we first evaluate the fitness of each individual in the population by calculating the sum of residuals between data and corresponding simulation results (‘cost’ C)

3 $$ C\left(\left\{\lambda, {\upgamma}_{2\mathrm{Gy}},\;\mathrm{P}\mathrm{S}{\mathrm{I}}_1,\;\mathrm{P}\mathrm{S}{\mathrm{I}}_2,\mathrm{P}\mathrm{S}{\mathrm{I}}_3,\mathrm{P}\mathrm{S}{\mathrm{I}}_4\right\}\right)={\sum}_{k=1}^4{\sum}_{t_{k,i}}\frac{{\left({V}_k\left({t}_{k,i}\right)-M\left(\left\{\lambda, {\upgamma}_{2\mathrm{Gy}},\mathrm{P}\mathrm{S}{\mathrm{I}}_1,\;\mathrm{P}\mathrm{S}{\mathrm{I}}_2,\mathrm{P}\mathrm{S}{\mathrm{I}}_3,\mathrm{P}\mathrm{S}{\mathrm{I}}_4\right\},{t}_{k,i}\right)\right)}^2}{V_{simulation,k}^2} $$

where *t*_*k,i*_ represents the time point when tumor volume *V*_*k*_ was measured for k*th* patient, and *M* is the simulated tumor volume at that time point. We then *select* the 50 % fittest individuals (i.e., parameter combinations) that have the smallest calculated cost (Eqn. 3), and discard unfit individuals. To maintain a constant population size N in subsequent generations, additional individuals are generated using *crossover* (25 %) and *mutation* (25 %) of survived *selected* individuals. For *crossover*, two survived *selected* individuals are chosen at random to generate a new individual ‘offspring’, by randomly *selecting* ‘parental’ parameter combinations (pairwise mixing). For *mutation*, a new individual is generated from a survived individual with either λ, γ_2Gy_ or PSI_*i*_ being modified randomly by changing its current value randomly up to ±10 %.

The 500 individuals after 500 iterations of the *selection, crossover*, and *mutation* procedure of the genetic algorithm (i.e., best sets of parameter combinations with the smallest sum of residuals between data and simulation results) is then refined with a trust-region-reflective algorithm (deterministic, gradient based optimization procedure) implemented in the MATLAB *lsqnonlin* function (MATLAB R2013b with Optimization Toolbox, The MathWorks Inc., Natick, MA), which uses a quadratic approximation for the minimized function in a neighborhood (trust region) around the current point. In order to avoid finding a parameter set yielding only local minimum in the fitness landscape we reiterated the whole fitting procedure 20 times and compared the resulting parameter sets.

### Alternative radiotherapy protocols

From the estimation of radiation-induced cell death, γ_2Gy_ we can approximate the radiosensitivity parameters α and β to derive γ_d_ for any dose *d* using the linear-quadratic model ${\upgamma}_d=1-{e}^{-\left(\upalpha \mathrm{d}+\upbeta {\mathrm{d}}^2\right)}$.

### Virtual patient cohort

We create a cohort of *n*=1,000 *in silico* virtual patients *P*_*i*_ for which we randomly assign tumor growth rate *λ*_*i*_ ∈ [*λ* * (1 − x %), *λ* * (1 + x %)] and radiation-induced cell death ${\upgamma}_2{{}_{\mathrm{Gy}}}_{{}_i}\in \left[{\upgamma_2}_{\mathrm{Gy}}\;*\;\left(1-\mathrm{x}\%\right),{\upgamma_2}_{\mathrm{Gy}}*\left(1+\mathrm{x}\%\right)\right]$ from uniform distributions, where x represents the level of uncertainty (width of the uniform distribution support). For each virtual patient we randomly assign tumor volume and PSI at beginning of treatment, and simulate tumor volume reduction after standard of care 2 Gy x 30 fractionation using Eqn. 1*.* We calculate the coefficient of determination, R^2^, to investigate how different degrees of uncertainty (value of x) impact the predictive power of PSI.

## Results

### Logistic tumor growth and radiation response model fits retrospective data

**Fig. 2:**
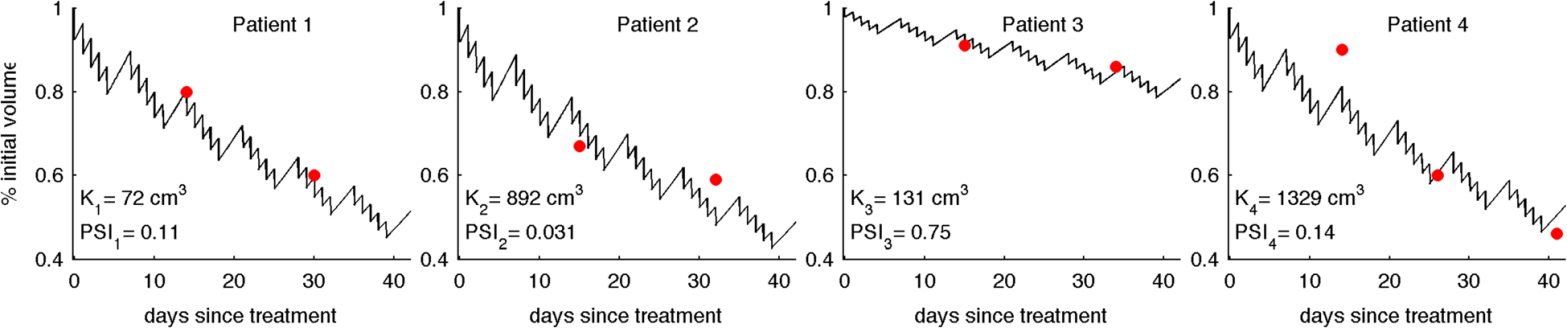
Genetic algorithm-derived fits of logistic tumor growth and radiation response model predicted curves (Eqn. 1; solid black lines) to 4 NSCLC patients data (red circles; [27]) with uniform growth rate λ=0.045 and radiation induced cell death γ_2Gy_=0.084, and patient-specific carrying capacities K_i_. PSI_i_: Proliferation Saturation Index for patient P_i_ at beginning of treatment (t=0)

The logistic tumor growth and radiation response model (Eqn. 1) fits retrospective longitudinal patient-specific data from the literature [27] with highest accuracy (R^2^=0.98) for λ=0.045 day^−1^ and γ_2Gy_=0.084 (Fig. 2). The estimate for λ suggests T_eff_=15.4 days, which indicates a cell loss factor of φ=50 % for T_POT_=7.7 days for lung adenocarcinoma [29]. Similar to oropharyngeal cancer cells, fast proliferating NSCLC cells are believed to have a relatively high α/β=20 [30–33]. With the α/β=20 approximated from literature, γ_2Gy_=0.084 suggests a radiosensitivity parameter α=0.0487 and thus S(2Gy)<91.6 %, which is similar to that reported for A549 if plated before irradiation (83.3 %; [34]) with additional consideration for senescence and transient cell cycle arrest.

Patient-specific carrying capacities are estimated at K={72, 892, 131, 1329} cm^3^, with pretreatment PSIs ranging from 0.03 (patient 2) to 0.75 (patient 3) with an average of 0.26±0.33 (Fig. 2). Interestingly, although the initial tumor volume of patient 4 is 25 times larger than that of patient 1, the reduction of tumor volume is comparable in both patients (53.4 % and 52.1 %) as their PSIs are similar (0.11 and 0.14). Patient 3, with an intermediate initial tumor volume but large PSI (0.75), has a significantly smaller reduction of tumor volume (20.4 %). Table 1 summarizes patient-specific tumor volumes V_i_, derived carrying capacities K_i_ and proliferation saturation indices PSI_i_, and other model parameter values. The minima obtained in each of the 20 independent iterations of the data fitting procedure were indistinguishable (standard deviation/mean = 6x10^−6^) with negligible differences between the estimated growth rates and radiosensitivities (λ=0.045±3x10^−5^ and γ_2Gy_ = 0.084±6x10^−5^). The differences in the estimated PSI_i_ values between independent data fitting iterations did not exceed 10 %.

**Table 1:** Summary of initial tumor volumes, parameters values and fractionation schemes used for four considered NSCLC patients [27]

	Initial tumor volume V(0), cm^3^	Carrying capacity, K, cm^3^	Proliferation saturation index, PSI, dimensionless	Growth rate, λ, day^−1^	Radiosensitivity, γ_2Gy_, dimensionless	Fractionation scheme
Patient 1	7.6	72	0.11	0.045	0.084	2 Gy x 30; daily at 9 am; weekend break
Patient 2	27.4	892	0.031			
Patient 3	97.7	131	0.75			
Patient 4	189.3	1329	0.14			

### Pretreatment PSI is a prognostic factor for radiation response

**Fig. 3:**
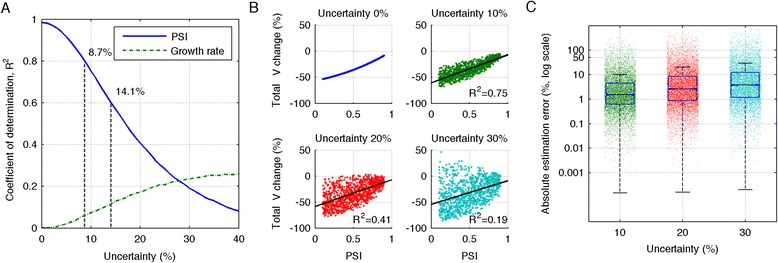
Pretreatment PSI as a prognostic factor. **a** Coefficient of determination, R^2^, for pretreatment PSI and growth rate λ as prognostic factors for tumor volume change after RT (2Gy x 30; q.d. 9 am) dependent on percentage of uncertainty in growth rate λ. **b** Predicted tumor volume change as a function of pretreatment PSI, dependent on uncertainty in growth rate λ. **c** Error in estimated patient-specific pretreatment PSI using Eq. 2 due to 5 % noise in measured tumor volumes and uncertainty in growth rate λ for *N*=10,000 independent simulations

For less than 8.7 % uncertainty in intrinsic tumor parameters, pretreatment PSI serves as a prognostic factor with a high coefficient of determination (R^2^>0.8; Fig. 3a). R^2^ falls below 0.6 for 14.1 % uncertainty in $\lambda$ and γ_2Gy_, but remains a better prognostic factor for radiation response than tumor growth rate for uncertainty in intrinsic tumor parameters up to around 25 %. Pretreatment PSI correlates inversely with tumor volume reduction during RT (Fig. 3b).

Reliable estimation of patient-specific pretreatment PSI must be achievable despite inter-patient variation in tumor growth rate $\lambda$, as well as limitation in radiological image resolution and noise in tumor volume measurements [35]. For each virtual patient *P*_*i*_ we reverse calculate exact tumor volume at diagnosis using Eqn. 1 (t-45 days; [26]), and introduce noise of ±5 % in the tumor volume measures at both diagnosis and treatment planning. The absolute error between exact pretreatment PSI and the proposed estimate using Eqn. 2 from noisy input data for different levels of uncertainty in tumor growth rate $\lambda$ is shown in Fig. 3c. For uncertainties less than 20 %, the absolute estimation error of pretreatment PSI is <10 % for more than 75 % of patients.

### Pretreatment PSI-dependent response to hyperfractionation

**Fig. 4:**
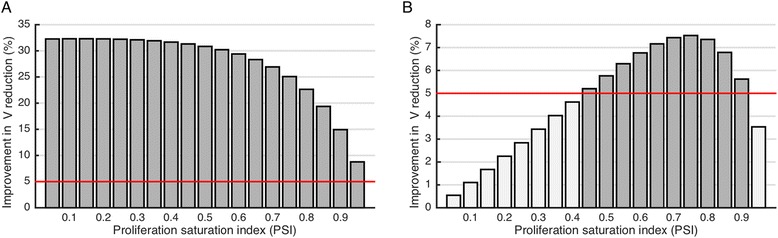
*In silico* comparison of altered fractionation regimes using parameters estimated for NSCLC patients. **a** Model predicted improvement in tumor volume reduction when comparing (1.2 Gy x 58; b.i.d. 9 am and 3 pm; BED=73.8 Gy) RTOG phase III hyperfractionation [36] to the standard of care (2 Gy x 30; q.d. 9 am; BED=66 Gy) as a function of proliferation saturation index (PSI). Red line at 5 % indicates statistical significance. **b** Model predicted improved tumor volume reduction when comparing (1.2 Gy x 58; b.i.d. 9 am and 3 pm) hyperfractionation to the daily doses with equal BED (2.21 Gy x 30; q.d. 9 am; BED=73.8 Gy) as a function of proliferation saturation index (PSI)

We consider tumors with fixed estimated radiosensitivity α=0.045 Gy-1, fixed α/β=20 Gy, and varying pre-treatment PSI. We simulate response to standard of care (2 Gy/fx x 30; q.d. 9 am; no weekend), and compare final tumor sizes to simulated responses to hyperfractionated treatment with 1.2 Gy/fx x 58; b.i.d. (9 am and 3 pm; no weekend), as prescribed in the experimental arm of an RTOG phase III trial in regionally advanced unresectable NSCLC [36]. Model simulations predict an average of 27.6 % improved tumor volume reduction after hyperfractionation for all PSI, due to the larger biologically effective dose (BED) (73.8 Gy_20_ vs. 66 Gy_20_ for standard of care) (Fig. 4a). To demonstrate which patients benefit from hyperfractionation, we compare the clinically applied hyperfractionation protocol to daily fractionation with equal BED (2.21 Gy x 30; q.d. 9 am; no weekend). An improvement in tumor volume reduction of >5 % is predicted for patients with intermediate PSI (0.45–0.9; Fig. 4b). This demonstrates that a patient-specific PSI may inform which patients are most likely to benefit from alternative radiation fractionation prior to clinical intervention.

## Conclusions

Despite inter-patient variability and the differences in tumor biology across disease sites, radiotherapy is conventionally fractionated at 180–200 cGy daily for 5–7 weeks. Current RT fractionation selection is based on average responses from large historical data sets and clinical trial cohorts without consideration of patient-specific characteristics. The potential uniqueness of each patient at diagnosis due to variation in tumor intrinsic properties such as radiosensitivity and host responses such as angiogenesis or immunoediting [14, 21], leads to the establishment of highly patient-specific circumstances, which can greatly affect clinical response. Tumors growing in tissues are faced with harsh biological and chemical conditions as well as physical forces, which all influence the maximum tumor volume that can be achieved in the current condition – the tumor carrying capacity. As a tumor population approaches its carrying capacity, overall proliferation rate saturates dependent on patient specific tumor history. As such, nominal tumor size alone is insufficient to predict growth dynamics.

It is conceivable that different tumor growth rates *in vitro* and *in vivo* are not primarily cell intrinsic; rather the impact of the *in vivo* environment may be the dominant mechanism that modulates cell behavior, which is absent when expanded in optimal *in vitro* conditions. The key feature of the patient-specific volume-to-carrying capacity ratio and the herein proposed proliferation saturation index, PSI, is a uniform cell growth rate as an intrinsic property that is modulated by host tissue conditions. This leads to different tumor population growth rates before, during and after radiation, including accelerated repopulation during radiotherapy. From two temporally separated radiological scans, routinely taken at diagnosis and treatment simulation, the change in individual *in vivo* tumor volume can be estimated and compared to the expected *in vitro* tumor growth, which allows for the estimation of the tumor environment-enforced patient-specific proliferation saturation index, PSI, using the analytic solution to the logistic growth model (Eqn. 1). If diagnostic images are unavailable, two subsequent images taken at later time points during therapy may be used to forecast the response to the remainder of the treatment schedule, and to adapt the protocol if necessary.

We introduced a patient-specific tumor carrying capacity in the logistic tumor growth model, and showed that simulations of tumor volume changes during RT using individual pretreatment PSI (equivalent to tumor volume-to-carrying capacity ratio) can reproduce historical radiation response data with high confidence (R^2^=0.98). Henceforth, *in silico* trials may be performed to predict which patients benefit from altered treatment protocols dependent on individual PSI and tumor volumes. The ability to forecast the response of individual tumors to different fractionations may pave the way for clinical trials to recommend either standard of care or alternative radiation fractionation on a per patient basis.

The presented model underestimates radiation-induced cell kill as non-proliferative cells are assumed to be completely radioresistant. Before consequential conclusions on alternative fractionations can be drawn, better approximations of radiation effects on the non-proliferative compartment have to be derived. We have refrained from such considerations in the present study in order to keep the number of unknown parameters small and thus preserve validity of the discussed concept. For simplicity we have limited our analysis to the effects of tumor environment on tumor properties and neglected variability in tumor-intrinsic properties. It is conceivable that future studies may integrate patient-specific molecular identifiers of intrinsic radiosensitivity [37], such as RSI [38, 39].

While simulation results using the concept of PSI fit retrospective data with high confidence, additional caution is warranted. The tumor carrying capacity is a dynamic entity that is unlikely to remain constant during the course of radiotherapy. Numerous biological processes such as vascular density, neovascularization, or immune surveillance contribute to variation in the carrying capacity, and the effects of radiation protocols on each of these processes individually and in combination are yet to be fully understood. Whilst high-resolution longitudinal measurements of contributors to carrying capacity, including those presently unidentified, are elusive, frequent tumor volume measurements during fractionated radiotherapy will provide patient-specific data to further fit the tumor growth model. It may also help us develop models that can form the basis for adaptive radiation therapy, which are greatly needed. For example, insights into the evolution of PSI during a course of therapy have the potential to help us understand the predictability of response. With a better understanding of the evolution of such dynamic factors as tumor response and PSI during the initially prescribed radiation protocol, different protocols could be simulated and the treatment protocol dynamically adapted to hopefully provide better outcomes. This will be the subject of future investigations.

## Abbreviations

PSI: Proliferation saturation index
V: Tumor volume
K: Tumor carrying capacity
RT: Radiotherapy
T_POT_: Potential doubling time
T_eff_: Effective doubling time
